# Beyond Mendelian Inheritance: Genetic Buffering and Phenotype Variability

**DOI:** 10.1007/s43657-021-00030-1

**Published:** 2021-12-27

**Authors:** Andrea Rossi, Zacharias Kontarakis

**Affiliations:** 1 Genome Engineering and Model Development Lab (GEMD), IUF-Leibniz Research Institute for Environmental Medicine, 40225 Düsseldorf, Germany; 2 Genome Engineering and Measurement Laboratory (GEML), Eidgenössische Technische Hochschule (ETH) Zurich, Zurich, Switzerland; 3 Functional Genomics Center Zurich of ETH Zurich, University of Zurich, 8093 Zurich, Switzerland

**Keywords:** Genetic compensation, Transcriptional adaptation, Modifiers, Phenotypes, Phenotypic plasticity

## Abstract

Understanding the way genes work amongst individuals and across generations to shape form and function is a common theme for many genetic studies. The recent advances in genetics, genome engineering and DNA sequencing reinforced the notion that genes are not the only players that determine a phenotype. Due to physiological or pathological fluctuations in gene expression, even genetically identical cells can behave and manifest different phenotypes under the same conditions. Here, we discuss mechanisms that can influence or even disrupt the axis between genotype and phenotype; the role of modifier genes, the general concept of genetic redundancy, genetic compensation, the recently described transcriptional adaptation, environmental stressors, and phenotypic plasticity. We furthermore highlight the usage of induced pluripotent stem cells (iPSCs), the generation of isogenic lines through genome engineering, and sequencing technologies can help extract new genetic and epigenetic mechanisms from what is hitherto considered ‘noise’.

## Introduction

The simplistic one-to-one relationship between genes and traits provides an excellent framework for understanding the core concepts of genetics, but it is not always a simple one-to-one relationship. For instance, even ‘identical twins’ who are raised together are never truly identical (Jonsson et al. 2021).

On one hand, the dominant or recessive genetic pattern of inheritance described by Mendel represents the extremes of a spectrum of states. On the other hand, this spectrum is (1) a function of allele strength, all mutant alleles are not equal; (2) genetic background, other genes influence sensitivity or resistance to a certain mutation; and (3) their interaction with the environment, a phenomenon known as phenotypic plasticity (West-Eberhard 1989).

Thus, the relationship between the genotypes of an organism and its phenotypes is complex. This complexity was illustrated by genetic studies of monogenic disorders in humans where the same mutation can produce different phenotypes (Quarton et al. 2020). Examples include cystic fibrosis (CF), Duchenne muscular dystrophy (DMD), Marfan syndrome and beta thalassemia, which are all monogenic disorders, but display wide phenotypic variability (Amaral 2015; Cao and Galanello 2010; Carter 1977; Lovering et al. 2005).

Nevertheless, a question that many scientists have been trying to answer is ‘What causes this phenotypic variability at the molecular level?’.

One part of the answer has started to unravel more than a century ago by the famous work of Calvin Bridges on modifier genes in *Drosophila melanogaster* (Bridges 1919). Modifier genes have the ability to influence the penetrance, dominance, expressivity, and pleiotropy of a phenotype (Nadeau 2001). Nowadays, the evidence for the action of modifier genes is extensive, both in humans and model organisms, and corresponding studies have shown that their effects on the phenotypic presentation of disease-causing variants can be subtle or profound (Cutting 2010; Nadeau 2001).

A typical example of a modifier gene is represented by the *APC*^*−/*+^ (adenomatous polyposis coli) mouse model, a murine counterpart of human *FAP* (familial adenomatous polyposis) (Moser et al. 1990). These mice display a wide phenotypic variation, for instance, the number of polyps, depending on the genetic background (Moser et al. 1990). By linkage analysis, a modifier gene, originally named *Mom1* (Modifier of Min-1) in the *APC*^*−/*+^ mouse model was mapped to the distal part of chromosome 4 (Dietrich et al. 1999). *Mom1* was able to explain 50% of the genetic variance in polyp number (Nadeau 2001). As at to date, many modifier genes have been described for different diseases including retinitis pigmentosa, CF, DMD, Bardet-Biedl syndrome, Marfan syndrome, Rett syndrome, Neurofibromatosis, Thalassemia, etc. (Collacoa and Cutting 2008; Dietz et al. 1994; Lobo 2008; Nadeau 2001).

Some modifier genes are inherited as genetic background, while others can be activated through different compensatory mechanisms (Hartman et al. 2001; Hilgert et al. 2009; Rossi et al. 2015; Rutherford 2000; Whitacre and Bender 2010).

Genetic buffering mechanisms such as genetic compensation and transcriptional adaptation can trigger the expression of modifier genes and have been proposed as mechanisms to explain apparent genotype to phenotype discrepancies (El-Brolosy et al. 2019; Lewontin 1974; Rossi et al. 2015; Wagner and Zhang 2011). By buffering the effects of deleterious genetic variations, cells can modify the outcome phenotype in different ways and at different levels (Rossi et al. 2015; El-Brolosy and Stainier 2017).

## Genetic Redundancy and Genetic Compensation

Eukaryotic cells show surprising robustness against internal and external perturbations, which can be partially attributed to functional redundancy established after small-scale and whole genome duplication events throughout evolution (Kuzmin et al. 2021; Lynch and Conery 2000).

After their duplication, genes initially gain redundant copies that can further evolve via processes such as non-functionalization, neofunctionalization or subfunctionalization into pseudogenes, genes with new biochemical functions or genes retaining part of the ancestral functions, respectively (Lundin 1999; Kuzmin et al. 2021). The presence of gene copies that retain some of the ancestral activity can provide a certain level of genetic redundancy that support genetic robustness (Ihmels et al. 2007; Kafri et al. 2006; Kuzmin et al. 2021; Li et al. 2010). The evolution of genetic robustness by duplication is followed by a neutral mode characterized by the loss of backup capacity, that is proportional to the divergence time. In the meantime, natural selection might act on a few pairs to maintain their long-term backup capacity, which are slowly evolving and now co-clustered in the same protein complexes and tend to interact with similar partners (Kuzmin et al. 2021). This can be defined as cellular robustness or genetic compensation arising from genetic redundancy (Stelling et al. 2004; Wagner 2005). The budding yeast *S. cerevisiae* has been a prime example of studying duplicated genes and redundancy (Goffeau et al. 1996). From a set of 201 duplicate gene pairs, 69 (34%) were found to be at least partially redundant (Dean et al. 2008). Furthermore, 49 of those redundant genes (71%) were synthetically lethal, indicating that their duplication partners could be compensating gene loss in the single mutants through functional redundancy (Dean et al. 2008).

In contrast to genetic redundancy, genetic compensation refers to an active mechanism where a deleterious mutation triggers the expression of modifier genes and does not develop the expected harmful phenotypes (Hartman et al. 2001; Rutherford 2000; Whitacre and Bender 2010). How genetic compensation be triggered is not fully understood, and different mechanisms seem to be involved. In general, genetic compensation seems to be achieved through protein feedback loops and to be independent of the types of mutation (Deconinck et al. 1997). A well-known example of genetic compensation is related to Dystrophin (DMD), a scaffold protein that links the actin cytoskeleton to the extracellular matrix (Blake et al. 2002; Nowak and Davies 2004). The absence of Dystrophin causes an X-linked genetic disorder, a devastating hereditary childhood disease characterized by progressive muscle degeneration, loss of ambulation in adolescence, and cardiopulmonary failure leading to the death of DMD patients during the third decades of their lives (Blake et al. 2002). *mdx* mice have been widely used to model DMD despite showing a milder phenotype compared to their human counterparts (Deconinck et al. 1997; Spitali et al. 2013). In these mice, an overall upregulation and sarcolemma recruitment of a modifier gene named *Utrophin* (*UTRN*) in the skeletal muscles and other tissues has been well described (Deconinck et al. 1997). A similar compensatory mechanism effect via UTRN upregulation has been suggested in some DMD patients, who also show higher levels of UTRN in their muscles and exhibit milder symptoms (Janghra et al. 2016; Nowak and Davies 2004; Kleopa et al. 2006). The mechanism that underlies UTRN upregulation is mediated, at least in part, by a protein feedback loop. The Dystrophin-associated protein complex (DAPC) has been shown to play both mechanical and nonmechanical roles in stabilizing the sarcolemma and protecting the muscle cells from contraction-induced damage. Thus, *DMD* mutations destabilize the DAPC and produce muscle weakness and muscular dystrophy (Ehmsen et al. 2002). The myogenic Akt (protein kinase B) signaling (Peter et al. 2009) family can be activated by numerous extracellular stimuli, an example being changes in the composition of extracellular matrix. In mouse models of dystrophy, due to the instability of the DAPC, signals are transduced by tyrosine kinase receptors and integrins followed by a subsequent cascade of Akt signaling that leads to the upregulation of UTRN (Peter et al. 2009). It is interesting to note that Integrin α7β1 is strongly upregulated in the *mdx* mice, and that this expression change has been linked to the reduction of the dystrophic phenotype and the partial restoration of viability in dystrophic mice (Lowell and Mayadas 2012). Other examples of genetic buffering via protein feedback loops include Anthrax toxin receptor 1 (ANTXR1), Lamins, Filamins, Emerin and other mechanosensing molecules (Cheng et al. 2019; Razinia et al. 2011; Shemesh et al. 2005).

## Transcriptional Adaptation

Transcriptional adaptation (TA) is a more recent example of ‘active’ genetic buffering where the presence of premature termination codons (PTCs) in engineered mutant alleles has been reported to correlate with cases of genotype–phenotype discrepancies (El-Brolosy et al. 2019; Ma et al. 2019; Rossi et al. 2015; Serobyan et al. 2020).

TA was initially described in zebrafish while analyzing discrepancies between antisense technology (morpholino oligos) and genetic engineering-based models for vascular development (Rossi et al. 2015). Following extensive discussion in the zebrafish field about the shift from antisense reagents to the newer genome engineering tools, a meta-analysis on the accumulating engineered zebrafish mutants lacking a phenotype had revealed a poor correlation between morpholino induced and genetic mutants, raising concerns about off-target effects (Kok et al. 2015; Schulte-Merker and Stainier 2014; Stainier et al. 2015). Knockdown of a protein product by inhibiting mRNA translation produced strong vascular phenotypes, in contrast to engineered mutants of the underlying gene, which developed a functional vascular system and were viable and fertile (Rossi et al. 2015). Unlike in cases of weak mutant alleles or unspecific effects of the antisense reagents used, mutant embryos were also resistant to knockdown, indicating that genetic rewiring had allowed them to develop through alternative avenues (Rossi et al. 2015). This result also argues against genetic redundancy, whereby the presence of redundant genes or pathways requires their combined inactivation to uncover a phenotype (Rossi et al. 2015).

In the same study, transcriptional and proteomic comparisons of the knockdown and knockout states lead to the identification of a group of modifier genes that were upregulated in the latter. Introducing some of those candidates could partially protect from the knockdown effects, indicating that these genes could replace the function of the mutated gene (Rossi et al. 2015). These results also reinforced the hypothesis that silent potential within the genome can be mobilized to overcome genetic insults (Kontarakis and Stainier 2020). Failure to do so can lead to disease, while successfully activating compensatory mechanisms, such as transcriptional adaptation could ameliorate disease symptoms (Kontarakis and Stainier 2020). Many reports involving zebrafish have since associated mild phenotypes to transcriptional adaptation, but only some have provided experimental evidence of such relationship (Kontarakis and Stainier 2020). A good example is the analysis of *actc1b *(Actin alpha cardiac muscle 1b) mutants, showing mild muscle defects and resistance to *actc1b* MO injection (Sztal et al. 2018). The authors showed the upregulation of *actc1a* (Actin Alpha Cardiac Muscle 1) and suggested that this paralogue served as a functionally redundant gene in *actc1b* mutants. Similarly, *nid1a* (Nidogen 1a) mutant zebrafish were reported to lack the short body phenotype exhibited by *nid1a* morphants (Zhu et al. 2017). The increased expression of *nid1b *(Nidogen 1b) and *nid2a* (Nidogen 2a) in *nid1a* mutants but not morphant zebrafish indicated that a transcriptional adaptation response was at play (Zhu et al. 2017). The authors were able to uncover the short body phenotype in *nid1a* mutants through *nid1b *and *nid2a* morpholino knockdown experiments. This result provided further support of the functional compensation provided by *nid1b *and *nid2a* activation in *nid1a* mutants.

How the transcriptome is modulated falls into the general field of gene expression regulation, but experimental support of a mechanism that initiates TA has been lacking (El-Brolosy and Stainier 2017). Two independent studies recently showed that in zebrafish and mouse models of transcriptional adaptation, the mutant mRNA serves as a signal to induce transcription of the adapting genes (El-Brolosy et al. 2019; Ma et al. 2019). This finding points to a number of possible mechanisms, including degradation via RNA quality control mechanisms, such as the nonsense mediated decay (NMD) (Brogna and Wen 2009). Nonsense-mediated decay represents only one form of such cellular pathways. The recent zebrafish data support a parallel pathway where the premature termination codon (PTC) containing mRNA is “saved” from degradation and repurposed as a regulator of transcription (Ma et al. 2019).

Processing of the repurposed mRNA or mRNA fragment, their transportation into the nucleus and the forming of transcriptional regulation units involve many currently unknown players. Both the complexity and conservation of this phenomenon have been highlighted in a report of transcriptional adaptation in *C. elegans* (Serobyan et al. 2020). A small candidate RNAi screen revealed players in RNA metabolism and transport that can influence the upregulation of adapting genes, and the resulting phenotypes. Taken together, small differences in seemingly housekeeping pathways could potentially affect the perceived strength of mutant alleles, adding an interesting and underexplored parameter to consider in phenotypic analyses (Kontarakis and Stainier 2020).

## Phenotypic Plasticity

It has been repeatedly proposed that another potential source of phenotypic variation in many monogenic diseases is the exposure to environmental factors or stressors (Gallati 2014). In this context, phenotypic variability, in which one genotype can produce more than one phenotype, when exposed to different forms of environmental stress, has been defined as phenotypic plasticity (Klingenberg 2019; Price et al. 2003; Via and Lande 1985).

Exposure of patients to different environmental conditions has been repeatedly proposed as an important factor which contributes to phenotypic plasticity in monogenic diseases (Gallati 2014). Clinical and basic science data suggest that non-genetic, i.e., exposomal factors (including environmental, life-style and dietary factors) might affect modifier gene expression and thereby contribute to the development of phenotypic plasticity (Cutting 2010; Cuvertino et al. 2017; Genin et al. 2008; Groman et al. 2002; Kleopa et al. 2006; Medici and Weiss 2017; Spiegler et al. 2018; Tummler 2019). The description and characterization of at least two genetic compensation pathways by which modifier gene expression can be regulated has provided one mechanistic explanation for phenotypic variability (El-Brolosy et al. 2019; Ma et al. 2019; Deconinck et al. 1997). One common denominator through which such factors could work is the generation of oxidative stress (Allen and Tresini 2000). An example that illustrates the impact of oxidative stress on clinical severity is the identification of single nucleotide polymorphisms in the glutathione pathway, which affect bacterial colonization and lung inflammation in patients suffering from cystic fibrosis (CF) (Marson et al. 2014). CF is a common, life limiting monogenic disease, which typically manifests as progressive bronchiectasis and recurrent sinopulmonary infections. After CFTR (cystic fibrosis transmembrane conductance regulator) was described in 1989, it has become increasingly evident that modifier genes and environmental factors play substantial roles in determining the severity of diseases (Collacoa and Cutting 2008; Maiuri et al. 2017). Analysis of siblings and twins with identical *CFTR* genotypes show different disease severity, strongly indicating that environmental factors play significant roles in determining the severity of CF (Mekus et al. 2000). Apart from phenotypic variation associated with oxidative stress, histone deacetylase (HDAC) inhibitors were proposed to be beneficial for CF patients (Angles et al. 2019). Interestingly, human bronchial epithelial cells exposed to environmental stress, including diesel exhaust particles, display increased *HDAC6* mRNA expression levels (Lin et al. 2020). HDAC’s mechanism of action in CF patients is not known, but HDAC molecules control transcriptional regulation and can interact with the ribosome, thus potentially controlling the expression of modifier genes.

Nevertheless, scientific evidence supporting the role of environmental stressors on phenotype variability are mainly circumstantial in nature, and the molecular mechanism(s) underlying phenotypic plasticity are currently not fully understood (Murren et al. 2015).

An intriguing possibility is that environmental stressors indirectly affect genetic buffering and modifier gene expression by interfering with RNA quality control pathways (e.g., NMD) (Nickless et al. 2017). In support of this hypothesis, it has been shown that environmental stress, including oxidative stress, suppresses NMD and affects cells by stabilizing NMD targeted gene expression (Usuki et al. 2019). In such a scenario, it can be postulated that the cells of a patient carrying a certain disease (premature stop codon mutation) would be unable to activate a compensatory machinery or enhance the expression of modifier genes and, therefore, should display a more severe phenotype. It is worth noting that CF patients that carry G542X, R553X, S1255X, W1316X CFTR PTC mutations show a severe mutant mRNA decrease, but display a mild but variable lung phenotype. In contrast, CF patients that carry R1162X, W1282X CFTR PTC mutations, in which mRNA stability is not affected, display a severe lung phenotype (Ferec et al 2012).

Cerebral cavernous malformations (CCM) are enlarged vascular lesions that consist of closely clustered, abnormally dilated and leaky capillary caverns that affect up to 0.2% of the general population (Choquet et al. 2015; Mouchtouris et al. 2015; Salman et al. 2008). A subset of mutation types have been identified within the three known *CCM* genes, which should allow for a better phenotype to genotype correlation and characterization (Choquet et al. 2015; Morrison and Akers 2003). Interestingly, the wide variability in phenotypes seen amongst different patients carrying the same mutation strongly suggests the influence of additional genetic and/or environmental components (Morrison and Akers 2003; Shenkar et al. 2015; Spiegler et al. 2018). In support of this, around 25% of people with cavernous malformations in the brain never have symptoms (Taslimi et al. 2016). Mouse models for cerebral cavernous malformations include *Ccm1* and *Ccm2* endothelial cell specific knockout mice (Boulday et al. 2011; McDonald et al. 2011). In these models, vascular malformations are seen at around postnatal day 6 (Tang et al. 2017).

Interestingly, it was noted that following a change in vivarium, *Ccm1* and *Ccm2* endothelial cell specific KO mice did not develop a severe phenotype (minimal to no hindbrain lesions). Further analysis revealed that a key role for the formation of CCM lesions was mediated by the gut microbiome through the activation of the Toll-like receptor 4 (TLR4) (Tang et al. 2017). Germ-free mice were protected from CCM formation and even a single course of antibiotics permanently alters CCM susceptibility in mice (Tang et al. 2017). Gene–environment interactions and phenotype variability have been described for other diseases but the molecular mechanisms underlying it are still not fully understood. Environmental factors, like exposure to toxic chemicals and brain injury, but also nutrition, traffic air pollution and virus or bacterial infections, have long been linked to the phenotype variability in many diseases including Alzheimer's, dementia, autism and Parkinson (Ball et al. 2019; Dunn et al. 2019).

## Genome Editing, Induced Pluripotent Stem Cells (iPSCs), Isogenic Lines and Organoid Models

iPSCs and genome editing combined represent a cutting-edge toolset to model diseases and better understand the biological processes that are still unsolved or poorly understood (Ramachandran et al. 2021). In particular, the creation of isogenic cell lines represents a precise control for the genetic disease model of interest, especially in those genes where discrepancies between genotype and phenotypes have been described.

One noteworthy development is the production of organoids from engineered cells to model and recapitulate disease phenotypes in three-dimensional tissues (Kim et al. 2020; Lancaster et al. 2013). This strategy provides a framework for both disease modeling and regenerative medicine based on the synthetic reconstitution of tissues. An interesting perspective would be to study genetic buffering in different cell types that carry the same type of mutation. Interesting questions to answer are: is the genetic buffering triggered at the same level in different cell types? Or is it cell specific? How are modifier genes affected by environmental stressors? Do environmental stressors affect mRNA quality control pathways that have been linked to TA?

These are all questions that, if scientifically addressed, might lead to better treatments and the development of new therapies that focus on enhancing the expression of modifier genes rather than fixing the ‘broken’ mutated gene. 

In the last few years, iPSCs and genome editing have been exploited to model human diseases using proper controls (isogenic lines) (Jones et al 2017). For instance, correcting the cystic fibrosis transmembrane regulator sequence in patient-derived iPSCs produced corrected cells that differentiated into healthy mature airway epithelial cells in vitro (Crane 2015). A mutation in the *Presenilin 1* (*PSEN-1*) gene, which is responsible for the majority of familial cases of Alzheimer's disease (AD), was corrected in iPSCs generated from a 58-year-old patient (Pires et al. 2016). CRISPR–Cas9 gene editing was also used to correct a mutation in the *DMD* gene in patient-derived iPSCs (Min et al. 2019).

## Closing Remarks

Sequencing studies on the Icelandic population using Illumina short reads (Gudbjartsson et al. 2015) and Oxford Nanopore long reads (Beyter et al. 2021) pointed out several loss of function mutations without apparent phenotypes, and one possible explanation is the presence of compensatory mechanisms, such as modifier genes, in these individuals (Sulem et al. 2015).

Furthermore, the advent of Nanopore sequencing technology has brought significant advantages to the field. For instance, alternative splicing (AS), alternative transcription initiation (ATI), and alternative cleavage and alternative polyadenylation (APA) have been identified as major contributors of transcriptome diversity (Lee et al. 2021). While AS events can be quantified and annotated using NGS with good accuracy, it has been hard to deduce full-length splicing isoforms that contain a combination of these individual splicing events (Lee et al. 2021).

Thus, long-read sequencing offers now the ability to map full-length sequences and potentially identify complex splice isoforms with diverse unknown roles, potentially also in genetic compensation.

Epigenetic modifications are heritable phenotypic changes that do not involve alteration of the nucleotide sequence but play a key role in gene expression and are associated with many diseases. Despite their presence in the human genome and the role in gene expression, base modifications are often overlooked due to difficulties with their detection (Liu and Seki 2020).

Using Nanopore sequencing, researchers have identified 5-methylcytosine (5mC), 5-hydroxymethylcytosine (5hmC), N6-methyladenine (6 mA), and Bromodeoxyuridine (BrdU) modifications in DNA, and through direct RNA sequencing N6-methyladenosine (m6A) modification in RNA (Liu et al. 2019). Furthermore, detection of other natural or synthetic epigenetic modifications is also possible through base calling algorithms training, and it could shed light on phenotypic variability. It has been proposed that these DNA and RNA modifications play a role in different biological processes including the control of gene expression and possibly genetic compensation (Barbieri and Kouzarides 2020; Liu et al. 2019).

Finally, the accumulation and storage of genetic variation in phenotypically normal populations is possible through genetic buffering. Silent variations can produce phenotypic differences when the buffering threshold is crossed, at which point these differences become sensitive to selection. Evolution and regulation of the balance between evolutionary stasis and change are regulated by buffering mechanisms. Yet, little is known about how buffering mechanisms work and respond to environment stimuli, thereby influencing phenotypic variability within a population.

## Data Availability

No datasets were generated or analyzed during this study.

## References

[CR1] Allen RG, Tresini M (2000). Oxidative stress and gene regulation. Free Radic Biol Med.

[CR02] Amaral MD (2015). Novel personalized therapies for cystic fibrosis: treating the basic defect in all patients. J Intern Med.

[CR400] Angles F, Hutt DM, Balch WE (2019). HDAC inhibitors rescue multiple disease-causing CFTR variants. Hum Mol Genet.

[CR2] Ball N, Teo W, Chandra S, Chapman J (2019). Parkinson's disease and the environment. Front Neurol.

[CR3] Barbieri I, Kouzarides T (2020). Role of RNA modifications in cancer. Nat Rev Cancer.

[CR4] Beyter D (2020). Long read sequencing of 3,622 Icelanders provides insight into the role of structural variants in human diseases and other traits. Nat Genet.

[CR5] Blake DJ, Weir A, Newey SE, Davies KE (2002). Function and genetics of dystrophin and dystrophin-related proteins in muscle. Physiol Rev.

[CR6] Boulday G, Rudini N, Maddaluno L, Blécon A, Arnould M, Gaudric A, Chapon F, Adams RH, Dejana E, Tournier-Lasserve E (2011). Developmental timing of CCM2 loss influences cerebral cavernous malformations in mice. J Exp Med.

[CR401] Bridges CB (1919). The genetics of purple eye color in Drosophila. J Exp Zool.

[CR7] Brogna S, Wen J (2009). Nonsense-mediated mRNA decay (NMD) mechanisms. Nat Struct Mol Biol.

[CR04] Cao A, Galanello R (2010). Beta-thalassemia. Genet Med.

[CR05] Carter CO (1977). Monogenic disorders. J Med Genet.

[CR8] Cheng B, Liu Y, Zhao Y, Li Q, Liu Y, Wang J, Chen Y, Zhang M (2019). The role of anthrax toxin protein receptor 1 as a new mechanosensor molecule and its mechanotransduction in BMSCs under hydrostatic pressure. Sci Rep.

[CR9] Choquet H, Pawlikowska L, Lawton MT, Kim H (2015). Genetics of cerebral cavernous malformations: current status and future prospects. Neurosurg Sci.

[CR10] Collacoa JM, Cutting GR (2008). Update on gene modifiers in cystic fibrosis. Curr Opin Pulm Med.

[CR11] Crane AM (2015). Targeted correction and restored function of the CFTR gene in cystic fibrosis induced pluripotent stem cells. Stem Cell Rep.

[CR12] Cutting GR (2010). Modifier genes in Mendelian disorders: the example of cystic fibrosis. Ann N Y Acad Sci.

[CR016] Cuvertino S, Stuart HM, Chandler KE, Roberts NA, Armstrong R, Bernardini L, Bhaskar S, Callewaert B, Clayton-Smith J, Davalillo CH, Deshpande C, Devriendt K, Digilio MC, Dixit A, Edwards M, Friedman JM, Gonzalez-Meneses A, Joss S, Kerr B, Lampe AK, Langlois S, Lennon R, Loget P, Ma DYT, McGowan R, Des Medt M, O'Sullivan J, Odent S, Parker MJ, Pebrel-Richard C, Petit F, Stark Z, Stockler-Ipsiroglu S, Tinschert S, Vasudevan P, Villa O, White SM, Zahir FR, Woolf AS, Banka S, Study D (2017). ACTB loss-of-function mutations result in a pleiotropic developmental disorder. Am J Hum Genetics.

[CR13] Dean EJ, Davis JC, Davis RW, Petrov DA (2008). Pervasive and persistent redundancy among duplicated genes in yeast. PLoS Genet.

[CR14] Deconinck AE, Rafael JA, Skinner JA, Brown SC, Potter AC, Metzinger L, Watt DJ, Dickson JG, Tinsley JM, Davies KE (1997). Utrophin-dystrophin-deficient mice as a model for Duchenne muscular dystrophy. Cell.

[CR15] Dietrich WF, Lander ES, Smith JS, Moser AR, Gould KA, Luongo C, Borenstein N, Dove W (1999). Genetic identification of Mom-1, a major modifier locus affecting Min-induced intestinal neoplasia in the mouse. Cell.

[CR16] Dietz HC, Ramirez F, Sakai LY (1994). Marfan’s syndrome and other microfibrillar diseases. Adv Hum Genet.

[CR17] Dunn AR, O’Connell KMS, Kaczorowski CC (2019). Gene-by-environment interactions in Alzheimer’s disease and Parkinson’s disease. Neurosci Biobehav Rev.

[CR18] Ehmsen J, Poon E, Davies K (2002). The dystrophin-associated protein complex. J Cell Sci.

[CR19] El-Brolosy MA, Stainier DYR (2017). Genetic compensation: a phenomenon in search of mechanisms. PLoS Genet.

[CR20] El-Brolosy MA, Kontarakis Z, Rossi A, Kuenne C, Günther S, Fukuda N, Kikhi K, Boezio GLM, Takacs CM, Lai SL, Fukuda R, Gerri C, Giraldez AJ, Stainier DYR (2019). Genetic compensation triggered by mutant mRNA degradation. Nature.

[CR017] Ferec C, Scotet V, Beucher J, Corvol H (2012). Genetics and modifier genes, atypical and rare forms. Arch Pediatr.

[CR09] Gallati S (2014). Disease-modifying genes and monogenic disorders: experience in cystic fibrosis. Appl Clin Genet.

[CR015] Genin E, Feingold J, Clerget-Darpoux F (2008). Identifying modifier genes of monogenic disease: strategies and difficulties. Hum Genet.

[CR22] Goffeau A, Barrell BG, Bussey H, Davis RW, Dujon B, Feldmann H, Galibert F, Hoheisel JD, Jacq C, Johnston M, Louis EJ, Mewes HW, Murakami Y, Philippsen P, Tettelin H, Oliver SG (1996). Life with 6000 genes. Science.

[CR014] Groman JD, Meyer ME, Wilmott RW, Zeitlin PL, Cutting GR (2002). Variant cystic fibrosis phenotypes in the absence of CFTR mutations. N Engl J Med.

[CR24] Gudbjartsson DF, Helgason H, Gudjonsson SA, Zink F, Oddson A, Gylfason A, Besenbacher S, Magnusson G, Halldorsson BV, Hjartarson E, Sigurdsson GT, Stacey SN, Frigge ML, Holm H, Saemundsdottir J, Helgadottir HT, Johannsdottir H, Sigfusson G, Thorgeirsson G, Sverrisson JT, Gretarsdottir S, Walters GB, Rafnar T, Thjodleifsson B, Bjornsson ES, Olafsson S, Thorarinsdottir H, Steingrimsdottir T, Gudmundsdottir TS, Theodors A, Jonasson JG, Sigurdsson A, Bjornsdottir G, Jonsson JJ, Thorarensen O, Ludvigsson P, Gudbjartsson H, Eyjolfsson GI, Sigurdardottir O, Olafsson I, Arnar DO, Magnusson OT, Kong A, Masson G, Thorsteinsdottir U, Helgason A, Sulem P, Stefansson K (2015). Large-scale whole-genome sequencing of the Icelandic population. Nat Genet.

[CR26] Hartman JL, Garvik B, Hartwell L (2001). Principles for the buffering of genetic variation. Science.

[CR27] Hilgert N, Huentelman MJ, Thorburn AQ, Fransen E, Dieltjens N, Mueller-Malesinska M, Pollak A, Skorka A, Waligora J, Ploski R, Castorina P, Primignani P, Ambrosetti U, Murgia A, Orzan E, Pandya A, Arnos K, Norris V, Seeman P, Janousek P, Feldmann D, Marlin S, Denoyelle F, Nishimura CJ, Janecke A, Nekahm-Heis D, Martini A, Mennucci E, Toth T, Sziklai I, Del Castillo I, Moreno F, Petersen MB, Iliadou V, Tekin M, Incesulu A, Nowakowska E, Bal J, Van de Heyning P, Roux AF, Blanchet C, Goizet C, Lancelot G, Fialho G, Caria H, Liu XZ, Xiaomei O, Govaerts P, Gronskov K, Hostmark K, Frei K, Dhooge I, Vlaeminck S, Kunstmann E, Van Laer L, Smith RJ, Van Camp G (2009). Phenotypic variability of patients homozygous for the GJB2 mutation 35delG cannot be explained by the influence of one major modifier gene. Eur J Hum Genet.

[CR28] Ihmels J, Collins SR, Schuldiner M, Krogan NJ, Weissman JS (2007). Backup without redundancy: genetic interactions reveal the cost of duplicate gene loss. Mol Syst Biol.

[CR29] Janghra N, Morgan JE, Sewry CE, Wilson FX, Davies KE, Muntoni F, Tinsley J (2016). Correlation of utrophin levels with the dystrophin protein complex and muscle fibre regeneration in duchenne and becker muscular dystrophy muscle biopsies. PLoS ONE.

[CR30] Jones VC, Atkinson-Dell R, Verkhratsky A, Mohamet L (2017). Aberrant iPSC-derived human astrocytes in Alzheimer’s disease. Cell Death Dis.

[CR31] Jonsson H (2021). Differences between germline genomes of monozygotic twins. Nat Genet.

[CR32] Kafri R, Levy M, Pilpel Y (2006). The regulatory utilization of genetic redundancy through responsive backup circuits. PNAS.

[CR33] Kim J, Koo B-K, Knoblich JA (2020). Human organoids: model systems for human biology and medicine. Nat Rev Mol Cell Biol.

[CR34] Kleopa KA, Drousiotou A, Mavrikiou E, Ormiston A, Kyriakides T (2006). Naturally occurring utrophin correlates with disease severity in Duchenne muscular dystrophy. Hum Mol Genet.

[CR35] Klingenberg CP (2019). Phenotypic plasticity, developmental instability, and robustness: the concepts and how they are connected. Front Ecol Evol.

[CR36] Kok FO, Shin M, Ni CW, Gupta A, Grosse AS, van Impel A, Kirchmaier BC, Peterson-Maduro J, Kourkoulis G, Male I, DeSantis DF, Sheppard-Tindell S, Ebarasi L, Betsholtz C, Schulte-Merker S, Wolfe SA, Lawson ND (2015). Reverse genetic screening reveals poor correlation between morpholino-induced and mutant phenotypes in zebrafish. Dev Cell.

[CR37] Kontarakis Z, Stainier DYR (2020). Genetics in light of transcriptional adaptation. Trends Genet.

[CR38] Kuzmin E, Taylor JS, Boone C (2021). Retention of duplicated genes in evolution. Trends Genet.

[CR39] Lancaster MA, Renner M, Martin CA, Wenzel D, Bicknell LS, Hurles ME, Homfray T, Penninger JM, Jackson AP, Knoblich JA (2013). Cerebral organoids model human brain development and microcephaly. Nature.

[CR81] Lee V, Judd LM, Jex AR, Holt KE, Tonkin CJ, Ralph SA (2021). Direct nanopore sequencing of mRNA reveals landscape of transcript isoforms in apicomplexan parasites. mSystems.

[CR40] Lewontin RC (1974). The genetic basis of evolutionary change.

[CR41] Li J, Yuan Z, Zhang Z (2010). The cellular robustness by genetic redundancy in budding yeast. PLoS Genet.

[CR020] Lin H, Fu G, Yu Q, Wang Z, Zuo Y, Shi Y, Zhang L, Gu Y, Qin L, Zhou T (2020). Carbon black nanoparticles induce HDAC6-mediated inflammatory responses in 16HBE cells. Toxicol Ind Health.

[CR42] Liu Xu, Seki M (2020). Recent advances in the detection of base modifications using the Nanopore sequencer. J Hum Genet.

[CR43] Liu Q, Fang L, Yu G, Wang D, Xiao CL, Wang K (2019). Detection of DNA base modifications by deep recurrent neural network on Oxford Nanopore sequencing data. Nat Commun.

[CR44] Lobo I (2008). Same genetic mutation, different genetic disease phenotype. Nat Educ.

[CR01] Lovering RM, Porter NC, Bloch RJ (2005). The muscular dystrophies: from genes to therapies. Phys Ther.

[CR45] Lowell CA, Mayadas TN (2012). Overview-studying integrins in vivo. Methods Mol Biol.

[CR46] Lundin LG (1999). Gene duplications in early metazoan evolution. Semin Cell Dev Biol.

[CR47] Lynch M, Conery JS (2000). The evolutionary fate and consequences of duplicate genes. Science.

[CR07] Ma Z, Zhu P, Shi H, Guo L, Zhang Q, Chen Y, Chen S, Zhang Z, Peng J, Chen J (2019). PTC-bearing mRNA elicits a genetic compensation response via Upf3a and COMPASS components. Nature.

[CR011] Maiuri L, Raia V, Kroemer G (2017). Strategies for the etiological therapy of cystic fibrosis. Cell Death Differ.

[CR012] Marson FAD, Bertuzzo CS, Ribeiro AF, Ribeiro JD (2014). Polymorphisms in the glutathione pathway modulate cystic fibrosis severity: a cross-sectional study. BMC Med Genet.

[CR48] McDonald DA, Shenkar R, Shi C, Stockton RA, Akers AL, Kucherlapati NH, Kucherlapati R, Brainer J, Ginsberg MH, Awad IA, Marchuk DA (2011). A novel mouse model of cerebral cavernous malformations based on the two-hit mutation hypothesis recapitulates the human disease. Hum Mol Genet.

[CR402] Medici V, Weiss KH (2017). Genetic and environmental modifiers of Wilson disease. HandbClin Neurol.

[CR010] Mekus F, Ballmann M, Bronsveld I, Bijman J, Veeze H, Tummler B (2000). Categories of deltaF508 homozygous cystic fibrosis twin and sibling pairs with distinct phenotypic characteristics. Twin Res.

[CR49] Min YL (2019). CRISPR-Cas9 corrects Duchenne muscular dystrophy exon 44 deletion mutations in mice and human cells. Sci Adv.

[CR50] Morrison L, Akers A (2003) Familial cavernous hemangioma, familial cerebral cavernous angioma, familial cerebral cavernous malformation. GeneReviews

[CR51] Moser AR, Pitot HC, Dove WF (1990). A dominant mutation that predisposes to multiple intestinal neoplasia in the mouse. Science.

[CR52] Mouchtouris N, Chalouhi N, Chitale A, Starke RM, Tjoumakaris SI, Rosenwasser RH, Jabbour PM (2015). Management of cerebral cavernous malformations: from diagnosis to treatment. Sci World J.

[CR53] Murren CJ, Auld JR, Callahan H, Ghalambor CK, Handelsman CA, Heskel MA, Kingsolver JG, Maclean HJ, Masel J, Maughan H, Pfennig DW, Relyea RA, Seiter S, Snell-Rood E, Steiner UK, Schlichting CD (2015). Constraints on the evolution of phenotypic plasticity: limits and costs of phenotype and plasticity. Heredity.

[CR54] Nadeau JH (2001). Modifier genes in mice and humans. Nat Rev Genet.

[CR56] Nickless A, Bailis JM, You Z (2017). Control of gene expression through the nonsense-mediated RNA decay pathway. Cell Biosci.

[CR57] Nowak KJ, Davies KE (2004). Duchenne muscular dystrophy and dystrophin: pathogenesis and opportunities for treatment. EMBO Rep.

[CR06] Peter AK, Ko CY, Kim MH, Hsu N, Ouchi N, Rhie S, Izumiya Y, Zeng L, Walsh K, Crosbie RH (2009). Myogenic Akt signaling upregulates the utrophin-glycoprotein complex and promotes sarcolemma stability in muscular dystrophy. Hum Mol Genet.

[CR58] Pires C, Schmid B, Petraeus C, Poon A, Nimsanor N, Nielsend TT, Waldemar G, Hjermind LE, Nielsen JE, Hyttel P, Freude KK (2016). Generation of a gene-corrected isogenic control cell line from an Alzheimer’s disease patient iPSC line carrying a A79V mutation in PSEN1. Stem Cell Res.

[CR08] Price TD, Qvarnstrom A, Irwin DE (2003). The role of phenotypic plasticity in driving genetic evolution. Proc Biol Sci.

[CR03] Quarton T, Kang T, Papakis V, Nguyen K, Nowak C, Li Y (2020). Uncoupling gene expression noise along the central dogma using genome engineered human cell lines. Nucleic Acids Res.

[CR59] Ramachandran H, Martins S, Kontarakis Z, Krutmann J, Rossi A (2021). Fast but not furious: a streamlined selection method for genome-edited cells. Life Sci Alliance.

[CR60] Razinia Z, Baldassarre M, Bouaouina M, Lamsoul I, Lutz PG, Calderwood DA (2011). The E3 ubiquitin ligase specificity subunit ASB2α targets filamins for proteasomal degradation by interacting with the filamin actin-binding domain. J Cell Sci.

[CR62] Rossi A, Kontarakis Z, Gerri C, Nolte H, Hölper S, Krüger M, Stainier DY (2015). Genetic compensation induced by deleterious mutations but not gene knockdowns. Nature.

[CR63] Rutherford SL (2000). From genotype to phenotype: buffering mechanisms and the storage of genetic information. BioEsseys.

[CR64] Salman RAS, Berg MJ, Morrison L, Awad IA (2008). Hemorrhage from cavernous malformations of the brain: definition and reporting standards. Stroke.

[CR66] Schulte-Merker S, Stainier DY (2014). Out with the old, in with the new: reassessing morpholino knockdowns in light of genome editing technology. Development.

[CR67] Serobyan V, Kontarakis Z, El-Brolosy MA, Welker JM, Tolstenkov O, Saadeldein AM, Retzer N, Gottschalk A, Wehman AM, Stainier DY (2020). Transcriptional adaptation in *Caenorhabditis elegans*. Elife.

[CR68] Shemesh T, Geiger B, Bershadsky AD, Kozlov MM (2005). Focal adhesions as mechanosensors: a physical mechanism. PNAS.

[CR69] Shenkar R, Shi C, Rebeiz T, Stockton RA, McDonald DA, Mikati AG, Zhang L, Austin C, Akers AL, Gallione CJ, Rorrer A, Gunel M, Min W, De Souza JM, Lee C, Marchuk DA, Awad IA (2015). Exceptional aggressiveness of cerebral cavernous malformation disease associated with PDCD10 mutations. Genet Med.

[CR71] Spiegler S, Rath M, Paperlein C, Felbor U (2018). Cerebral cavernous malformations: an update on prevalence, molecular genetic analyses, and genetic counselling. Mol Syndromol.

[CR72] Spitali P, van den Bergen JC, Verhaart IEC, Wokke B, Janson AAM, van den Eijnde R, den Dunnen JT, Laros JFJ, Verschuuren JJGM, Hoen PA, Aartsma-Rus A (2013). DMD transcript imbalance determines dystrophin levels. FASEB J.

[CR73] Stainier DY, Kontarakis Z, Rossi A (2015). Making sense of anti-sense data. Dev Cell.

[CR75] Stelling J, Gilles ED, Doyle FJ (2004). Robustness properties of circadian clock architectures. 10.1038/ng.3243.

[CR76] Sulem P, Helgason H, Oddson A, Stefansson H, Gudjonsson SA, Zink F, Hjartarson E, Sigurdsson GT, Jonasdottir A, Jonasdottir A, Sigurdsson A, Magnusson OT, Kong A, Helgason A, Holm H, Thorsteinsdottir U, Masson G, Gudbjartsson DF, Stefansson K (2015). Identification of a large set of rare complete human knockouts. Nat Genet.

[CR77] Sztal TE, McKaige EA, Williams C, Ruparelia AA, Bryson-Richardson RJ (2018). Genetic compensation triggered by actin mutation prevents the muscle damage caused by loss of actin protein. PLoS Genet.

[CR79] Tang AT, Choi JP, Kotzin JJ, Yang Y, Hong CC, Hobson N, Girard R, Zeineddine HA, Lightle R, Moore T, Cao Y, Shenkar R, Chen M, Mericko P, Yang J, Li L, Tanes C, Kobuley D, Vosa U, Whitehead KJ, Li DY, Franke L, Hart B, Schwaninger M, Henao-Mejia J, Morrison L, Kim H, Awad IA, Zheng X, Kahn ML (2017). Endothelial TLR4 and the microbiome drive cerebral cavernous malformations. Nature.

[CR80] Taslimi S, Modabbernia A, Amin-Hanjani S, Barker FG, Macdonald RL (2016). Natural history of cavernous malformation. Systematic review and meta-analysis of 25 studies. Neurology.

[CR013] Tummler B (2019). Mild cystic fibrosis in carriers of two nonsense mutations—a case of genetic compensation response?. J Cyst Fibros.

[CR018] Usuki F, Yamashita A, Fujimura M (2019). Environmental stresses suppress nonsense-mediated mRNA decay (NMD) and affect cells by stabilizing NMD-targeted gene expression. Sci Rep.

[CR82] Via S, Lande R (1985). Genotype-environment interaction and the evolution of phenotypic plasticity. Evolution.

[CR83] Wagner A (2005). Distributed robustness versus redundancy as causes of mutational robustness. BioEssays.

[CR84] Wagner GP, Zhang J (2011). The pleiotropic structure of the genotype-phenotype map: the evolvability of complex organisms. Nat Rev Genet.

[CR85] West-Eberhard MJ (1989). Phenotypic plasticity and the origins of diversity. Annu Rev Ecol Syst.

[CR86] Whitacre J, Bender A (2010). Degeneracy: a design principle for achieving robustness and evolvability. J Theor Biol.

[CR87] Zhu P, Ma Z, Guo L, Zhang W, Zhang Q, Zhao T, Jiang K, Peng J, Chen J (2017). Short body length phenotype is compensated by the upregulation of nidogen family members in a deleterious nid1a mutation of zebrafish. J Genet Genom.

